# Berbamine Targets TNFAIP3: A Bioactive Compound Alleviates Oxidative Stress and Inflammation in the Comorbidity of Insomnia and Chronic Obstructive Pulmonary Disease Through Multi-Omics Integration

**DOI:** 10.3390/ijms262010227

**Published:** 2025-10-21

**Authors:** Xinliao Deng, Shuaiyu Jiang, Ziyi Liu, Xinyu Liu, Tao Lu, Xiaodan Liu

**Affiliations:** 1 School of Rehabilitation Science, Shanghai University of Traditional Chinese Medicine, Shanghai 201203, China; 2961@shutcm.edu.cn (X.D.); jshuaiy@163.com (S.J.); 2 School of Traditional Chinese Medicine, Shanxi Datong University, Datong 037009, China; 221002011619@sxdtdx.edu.cn (Z.L.); jinyeyou@hotmail.com (X.L.)

**Keywords:** chronic obstructive pulmonary disease, insomnia, mendelian randomization, comorbidity mechanisms, natural small molecule compounds

## Abstract

Chronic obstructive pulmonary disease (COPD) and insomnia are highly comorbid, yet their shared pathogenesis and therapeutic targets remain unclear. This study employed multidimensional approaches—including bidirectional Mendelian randomization (MR), transcriptomic analysis, weighted gene co-expression network analysis (WGCNA), and computational drug repositioning—to investigate causal relationships, shared pathways, and therapeutic strategies for COPD–insomnia comorbidity. MR analysis indicated that insomnia is a causal risk factor for COPD (OR = 2.04, 95% CI: 1.18–3.51; *p* = 0.011), with no reverse causality. Integrated transcriptomics of COPD (GSE148004) and insomnia (GSE208668) identified 230 co-dysregulated genes enriched in immune-inflammatory pathways (e.g., NF-κB signaling and cytokine response) and oxidative stress. Protein–protein interaction networks highlighted TNFAIP3 as a hub gene, confirmed by LASSO regression as a shared diagnostic biomarker. A co-expression network of 190 overlapping genes linked circadian disruption and airway inflammation. Drug repositioning nominated TNFAIP3-targeting agents, and molecular docking revealed high-affinity binding between berbamine and the TNFAIP3 OTU domain (ΔG = −9.25 kcal/mol). TNFAIP3 emerges as a dual regulator of inflammatory signaling and redox homeostasis. Our systems pharmacology approach bridges epidemiological causality and molecular mechanisms, supporting single-agent polypharmacology for COPD–insomnia comorbidity.

## 1. Introduction

Chronic obstructive pulmonary disease (COPD), characterized by persistent and progressive airflow obstruction, affects nearly 400 million people globally and is the third leading cause of death worldwide [1,2]. Sleep disorders frequently coexist with COPD and may worsen its course [3]. Poor sleep quality, measured by the Pittsburgh Sleep Quality Index (PSQI), is associated with an increased risk of COPD exacerbation [4,5], suggesting that sleep-focused interventions could reduce exacerbations and improve disease management [6,7].

Multiple studies confirm an epidemiological link between COPD and insomnia, showing a vicious cycle: insomnia raises the risk of acute exacerbations [8], while chronic COPD symptoms like nocturnal dyspnea worsen sleep [9]. This co-occurrence presents a complex clinical challenge, making treatment difficult. It not only increases patient burden but also complicates disease management [10].

The treatment of COPD typically follows clear guidelines; however, for patients with both COPD and insomnia, treatment outcomes are often suboptimal. During acute exacerbations of COPD, although systemic corticosteroids and antibiotics are widely recommended treatments, they do not effectively improve insomnia symptoms [11]. From a physiological perspective, chronic hypoxia and the release of inflammatory factors (such as tumor necrosis factor-α(TNF-α) and Interleukin-6(IL-6)) trigger oxidative stress, which exacerbates airway remodeling and disruption of the circadian rhythm through the NF-κB pathway [12,13]. From a psychological perspective, depression associated with COPD (with a prevalence of approximately 40%) reduces treatment adherence, while insomnia exacerbates pain sensitivity, forming a ‘pain–sleep disorder cycle.’ This makes the co-management and treatment of COPD and insomnia highly challenging, necessitating the identification of effective medications that can simultaneously address both conditions [10,14]. This study identifies TNFAIP3 as a key molecular target linking chronic obstructive pulmonary disease (COPD) and insomnia, and proposes berbamine as a potential therapeutic drug, filling the gap in understanding shared mechanisms and dual-treatment strategies for this comorbidity.

TNFAIP3, also known as A20, is a multifunctional protein that plays a key role in cellular signaling and immune regulation. Through its deubiquitinating enzyme activity, A20 modulates the ubiquitinated state of RIP1 and TRAF6, thereby influencing multiple cellular processes. The existing research indicates that A20 performs a crucial role in the regulation of inflammatory responses and apoptosis [15,16]. Firstly, A20 exerts a negative regulatory role in inflammatory responses by deubiquitinating RIP1 and TRAF6, thereby inhibiting the activation of the NF-κB signaling pathway [17,18]. Moreover, the role of A20 in immune regulation is also significant. The suppression of excessive immune responses during systemic fungal infections is achieved by the regulation of the C-type lectin receptor (CLR) signaling pathway by A20, thereby enhancing host survival [19]. This regulatory mechanism demonstrates that A20 not only functions in intracellular signaling but also holds significant importance in the overall immune response. TNFAIP3, a pivotal negative regulator, has been demonstrated to modulate inflammatory responses by suppressing the NF-κB signaling pathway, a process that is of considerable significance for the pathological progression of COPD [20]. In the pathophysiological mechanisms of COPD, the presence of insomnia adds to the complexity of the disease. Research indicates that insomnia is associated with an increased risk of acute exacerbations in COPD patients, potentially due to heightened inflammatory responses and exacerbated oxidative stress resulting from sleep disturbances [9]. Functional studies of TNFAIP3 in COPD with insomnia reveal its potential role in regulating inflammatory responses. In-depth investigation of TNFAIP3 may offer novel insights and therapeutic strategies for COPD and its associated complications. Future research should further elucidate the specific mechanisms of TNFAIP3 in COPD with insomnia, aiming to provide more precise targets for clinical interventions. Inflammatory mediators such as tumor necrosis factor alpha (TNFα) and lipopolysaccharide (LPS) play pivotal roles in immune responses and act as regulators of A20, modulating inflammatory reactions by inducing A20 (TNFAIP3) expression. Research indicates that TNFα and LPS significantly upregulate A20 expression, a process validated across multiple cell types [21,22].

The study aimed to investigate the shared molecular mechanisms and therapeutic targets between COPD and insomnia using a multidimensional approach, including Mendelian randomization, transcriptomic analysis, and computational drug repositioning, to identify causal relationships, key biomarkers, and potential dual-treatment strategies for this comorbidity. In summary, this study provides theoretical foundation for future research into the mechanisms and treatment strategies for COPD–insomnia comorbidity.

In the context of aging populations, the comorbidity of COPD and insomnia is particularly prevalent among the elderly, who often exhibit heightened vulnerability due to age-related declines in immune function, circadian rhythm disruption, and polypharmacy. Therefore, our study, while not exclusively focused on the elderly, holds significant implications for this demographic, which bears a disproportionate burden of both conditions.

## 2. Results

### 2.1. Mendelian Randomisation

**Figure 1:**
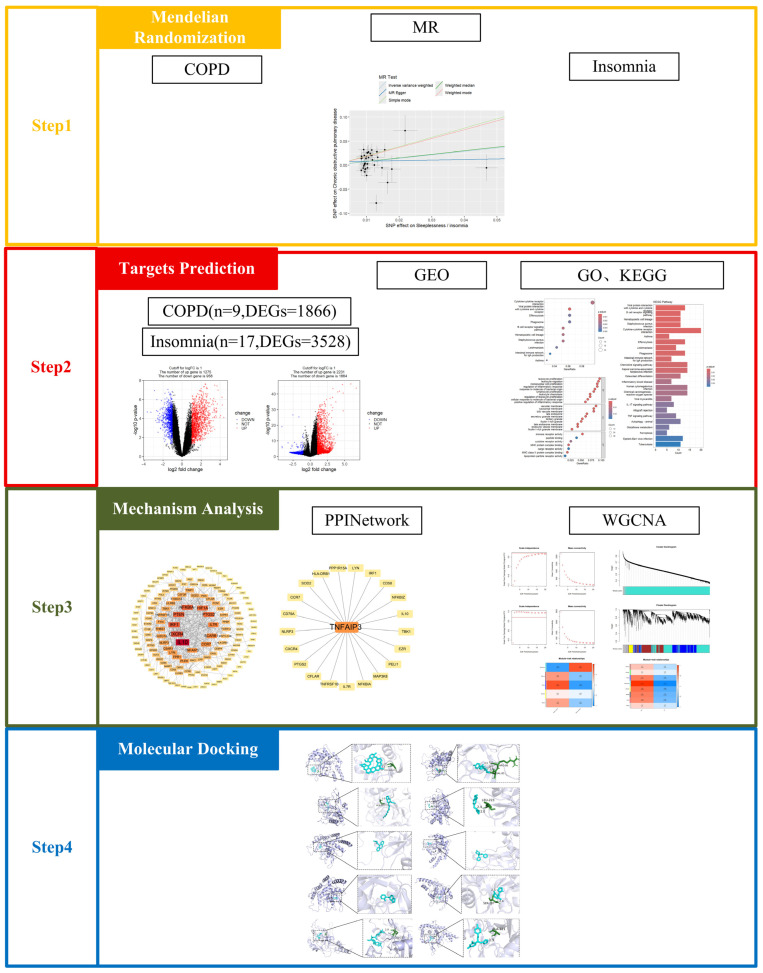
Flow chart of this study. This flow chart describes the design idea of the study, the experimental steps, including Mendelian randomization, target prediction, machine learning, molecular docking.

**Figure 2:**
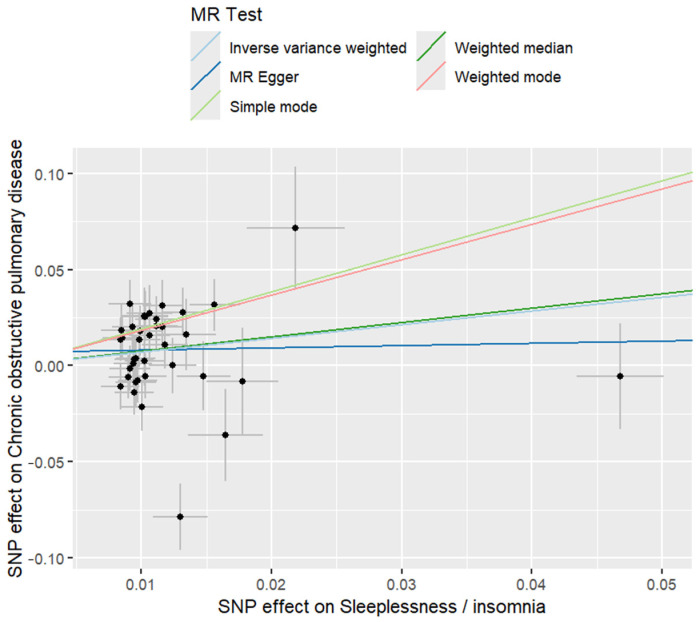
Two-way Mendelian randomization analysis Scatter plot of univariable MR model The *x*-axis represents snp effect on sleeplessness, while the *y*-axis represents snp effect on chronic obstructive pulmonary disease.

The bidirectional MR analysis revealed insomnia as a causal risk factor for COPD development, (heterogeneity-adjusted Cochran’s Q = 18.3, *p* = 0.24), 39 genome-wide significant insomnia-associated SNPs (mean F-statistic = 31.7) showed a positive causal effect (OR = 2.04, 95% CI = 1.18–3.51; *p* = 0.011). Sensitivity analyses confirmed the robustness of these results. The MR-Egger intercept showed no directional pleiotropy (intercept *p* = 0.38); the weighted median estimator maintained significance (OR = 1.91, 95% CI = 1.05–3.47), and the leave-one-out sensitivity demonstrated stability (maximum OR fluctuation = ±0.12). Reverse MR analysis of 11 COPD-associated characteristics (mean F = 29.4) showed null causality on insomnia risk (IVW, *p* = 0.41). Multivariable adjustment for smoking exposure had minimal effects on these associations (OR = 1.97, 95% CI = 1.14–3.39), following Bonferroni correction for bidirectional testing (α = 0.025). The unidirectional causal model suggests that insomnia severity escalates COPD susceptibility, whereas pulmonary impairment exhibits no neurological feedback (Figure 1 and Figure 2), (Figures S1 and S2).

### 2.2. Targets of COPD and Insomnia

**Figure 3:**
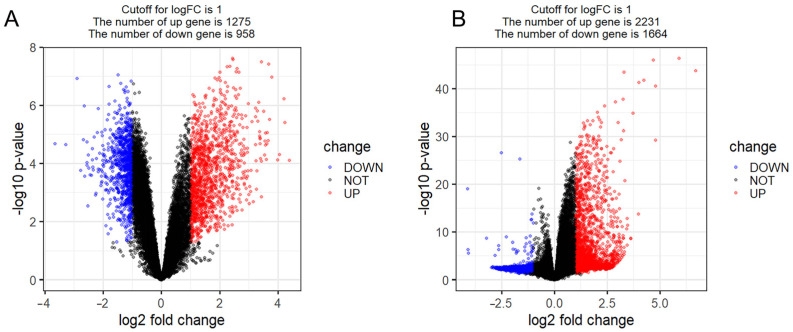
Enrichment analysis of 230 co-regulated genes for immune-related functions. Bubble size represents the number of enriched genes, and color depth indicates the level of significance. (**A**) Differentially expressed genes (DEGs) in the GSE148004 dataset (COPD vs. controls). (**B**) Differentially expressed genes (DEGs) in the GSE208668 dataset.

Transcriptomic profiling of the GSE148004 (COPD vs. controls: 1866 DEGs, 1275 upregulated and 958 downregulated; FDR < 0.05, fold-change > 1.5) and GSE208668 cohorts (insomnia vs. controls: 3528 DEGs, 2231 upregulated and 1664 downregulated; FDR < 1 × 10^−6^) revealed significant molecular convergence between disorders. We identified 367 co-dysregulated genes (230 conserved upregulation, fold-change > 1.8; 137 coordinated downregulation, fold-change < 0.6) at the COPD–insomnia intersection (Fisher’s exact test, Poverlap = 3.2 × 10^−12^), 230 of which are simultaneously regulated in COPD and insomnia (Figure 3 and Figures S3 and S4).

### 2.3. Functional Enrichment Analysis

**Figure 4:**
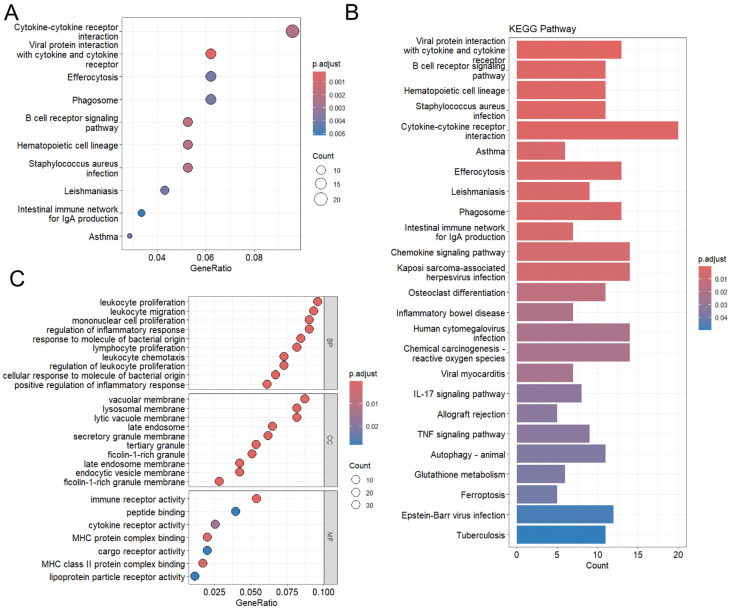
KEGG pathway and GO enrichment analysis of DEGs. (**A**) Scatter plot with point color (adjusted P) and size (count). (**B**) Bar plot showing the KEGG pathways. Bar length indicates count; color represents adjusted *p* value. (**C**) Heatmap showing the enriched GO terms in the BP, CC, and MF categories.

The GO and KEGG entries of the 230 co-regulated genes were mostly enriched and associated with immune response. The most enriched GO terms in the BP category included the positive regulation of cytokine production, leukocyte proliferation, leukocyte migration, monocyte proliferation, regulation of inflammatory response, response to molecules of bacterial origin, lymphocyte value-added, leukocyte chemotaxis, regulation of leukocyte value-added. In the CC category, the most enriched GO terms were vacuolar membrane, lysosomal membrane, lytic vacuole membrane, late endosome, secretory granule membrane, tertiary granule, ficolin-1-rich granule, and other pathways. MF was mainly enriched in immune receptor activity, MHC protein complex binding, and MHC class II protein complex binding. KEGG pathway analysis showed that the common gene set was associated with pathways related to viral protein interaction with cytokine and cytokine receptor, B cell receptor signaling, hematopoietic cell lineage, and Staphylococcus aureus infection. These pathways are closely related to immune responses, cell differentiation, and metabolic processes (Figure 4), (Figures S5 and S6).

### 2.4. Interaction Network Analysis

**Figure 5:**
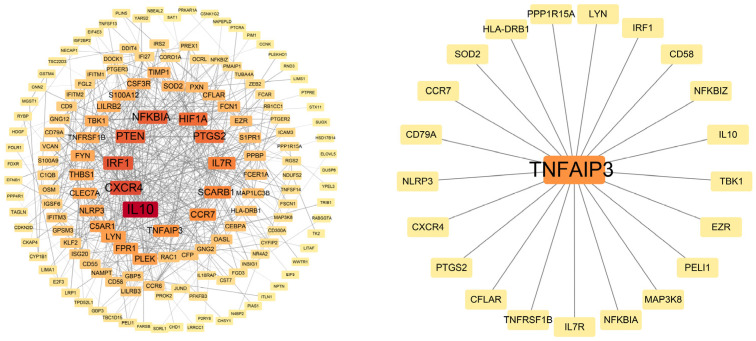
Protein–protein interaction network of genes co-expressed in COPD and insomnia. The node size reflects the gene’s degree value. Red represents the most central proteins, orange the more central proteins, and yellow the less relevant proteins.

From the 230 Co-Differentially Expressed Genes, a PPI interaction network was constructed, comprising 164 nodes and 1146 edges (Figure 5). The resultant network comprised 164 nodes (proteins) and 1146 edges (interactions) significantly enriched for physical and functional interactions. The average node size was 6.99.

### 2.5. Identification of Key Modules

**Figure 6:**
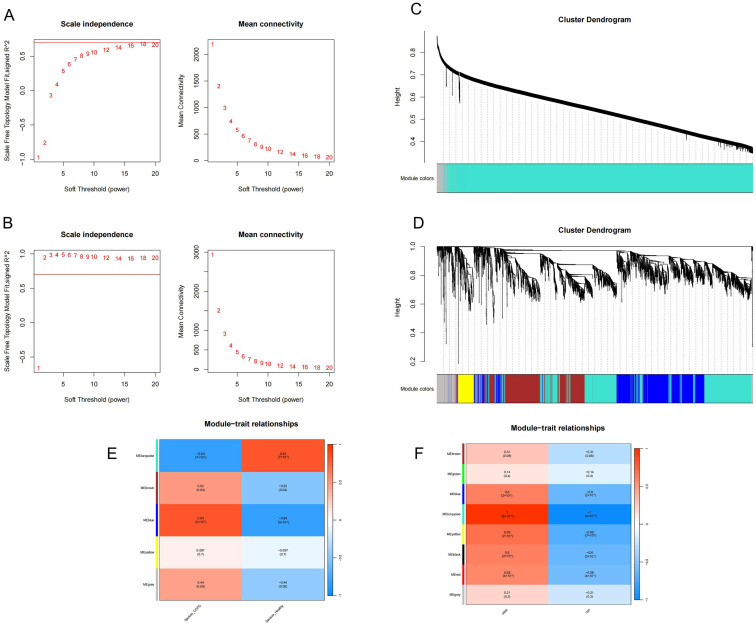
WGCNA-based analysis of COPD and insomnia co-expression modules. (**A**) Scale-free network topology analysis. (**B**) Soft-threshold power screening. (**C**) Gene co-expression clustering dendrogram. (**D**) Visualization of co-expression module features, with warm colors indicating high correlation. (**E**) Heatmap of co-expression module–phenotype correlation. Red indicates positive correlation, while blue indicates negative correlatio. (**F**) Heatmap of co-expression modules associated with insomnia phenotypes.

**Figure 7:**
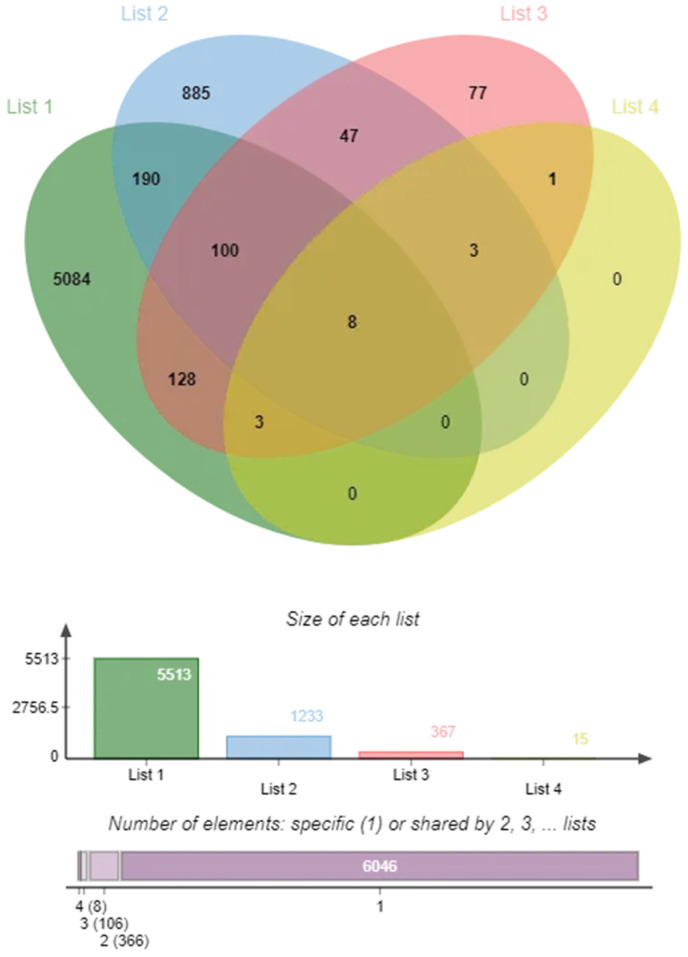
Overlap and size distribution of four datasets.

A total of 10 modules in the GSE148004 and GSE208668 datasets were identified. The heatmap shows 190 overlapping genes between the COPD and insomnia positive modules (Figure 6 and Figure 7), (Figures S7).

### 2.6. Biomarker Screening

**Figure 8:**
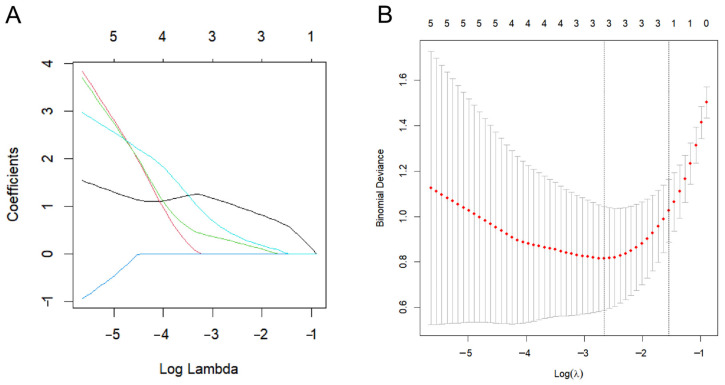
LASSO regression analysis for diagnostic biomarker identification in COPD and insomnia. (**A**) Coefficient shrinkage profiles (*y*-axis) vs. log Lambda (*x*-axis), with three non-zero features retained. (**B**) Binomial deviance curve (minimum at logλ = −1) for optimal parameter selection.

LASSO regression identified 1 common diagnostic biomarker gene for COPD and insomnia, namely TNFAIP3 (Figure 8, Table 1).

### 2.7. Drug Signatures

The drug repositioning analysis identified several potential therapeutic compounds targeting TNFAIP3, a key hub gene shared by COPD and insomnia. Berbamine emerged as the top candidate with the highest binding affinity (Combined score = 150,029.82, ΔG = −9.25 kcal/mol), followed by tolbutamide (Combined score = 148,283.12) and piperonyl butoxide (Combined score = 148,283.12). These compounds exhibited significant associations (*p* < 0.0013, Adjusted *p* < 0.013) and high odds ratios (OR > 19,975), suggesting strong mechanistic links to TNFAIP3 modulation (Table 2).

### 2.8. Molecular Docking Analysis

**Figure 9:**
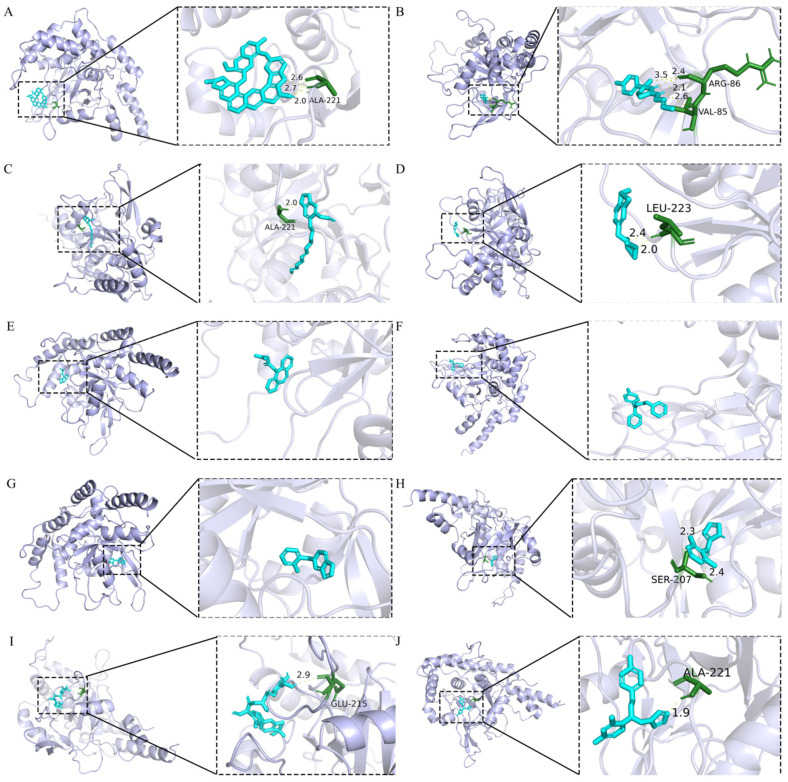
Molecular docking results between TNFAIP3 and berbamine, tolbutamide, piperonyl, fenthion, trimipramine, cloperastine, ticlopidine, clonidine, lasalocid, and econazole. (**A**) Molecular docking results between TNFAIP3 and berbamine. (**B**) Molecular docking results between TNFAIP3 and tolbutamide. (**C**) Molecular docking results between TNFAIP3 and piperonyl. (**D**) Molecular docking results between TNFAIP3 and fenthion. (**E**) Molecular docking results between TNFAIP3 and trimipramine. (**F**) Molecular docking results between TNFAIP3 and cloperastine. (**G**) Molecular docking results between TNFAIP3 and ticlopidine. (**H**) Molecular docking results between TNFAIP3 and clonidine. (**I**) Molecular docking results between TNFAIP3 and lasalocid. (**J**) Molecular docking results between TNFAIP3 and econazole.

To identify the potential modulators of TNFAIP3 (A20), a ubiquitin-editing enzyme critical for the regulation of NF-κB signaling, molecular docking studies were performed against its ovarian tumor (OTU) domain (PDB ID: 2VFJ), which mediates K63-linked deubiquitination of substrates, such as RIP1 and TRAF6. The binding free energies (ΔG) of 10 compounds were calculated and ranked in increasing order as follows: berbamine (−9.25 kcal/mol) > tolbutamide (−8.68 kcal/mol) > ticlopidine (−8.02 kcal/mol) > econazole (−7.1 kcal/mol) > cloperastine (−6.58 kcal/mol) > fenthion (−6.08 kcal/mol) > trimipramine (−5.98 kcal/mol) > clonidine (−5.76 kcal/mol) > piperonyl (−4.93 kcal/mol) > lasalocid (−1.43 kcal/mol) (Table 3 and Figure 9).

### 2.9. Immune Infiltration Landscape Reveals Shared Inflammatory Signature

**Figure 10:**
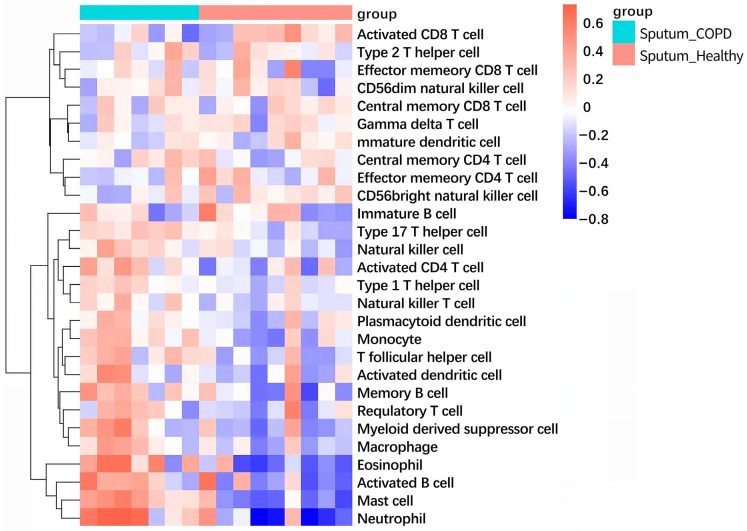
Immune cell infiltration landscape in COPD (GSE148004). The heatmap displays the relative abundance of 28 immune cell types in COPD patients compared to controls, quantified by single-sample GSEA (ssGSEA) normalized enrichment scores. Red indicates increased infiltration; blue indicates decreased infiltration.

**Figure 11:**
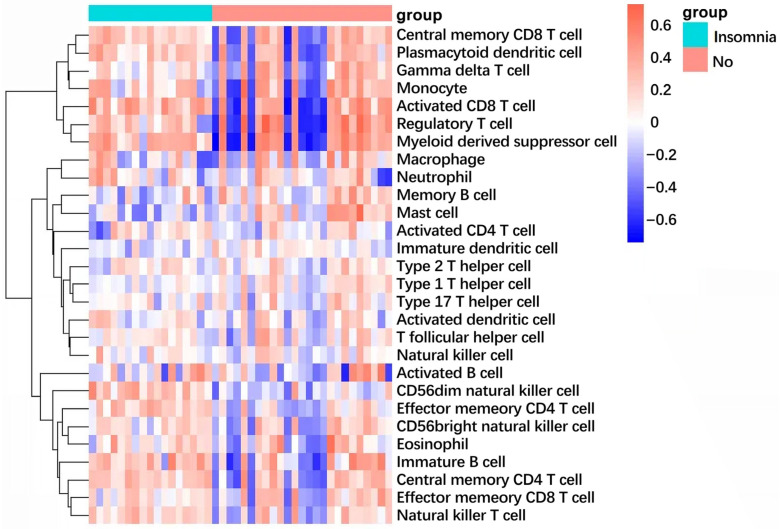
The heatmap illustrates the immune infiltration profile in insomnia subjects versus controls, derived from ssGSEA analysis.

The ssGSEA analysis uncovered significant alterations in immune cell infiltration patterns in both COPD and insomnia. In the COPD cohort (Figure 10), we observed a pronounced enrichment of innate immune cells, particularly neutrophils and macrophages, alongside a relative reduction in adaptive immune cells such as central memory CD4+ T cells and effector memory CD4+ T cells. Similarly, in the insomnia cohort (Figure 11), there was a consistent pattern of innate immune activation, with elevated infiltration of neutrophils, macrophages, and monocytes. Notably, both conditions showed decreased abundance of CD56 bright natural killer cells and certain T cell subsets, suggesting a convergent impairment of adaptive immunity. These findings provide cellular evidence for the shared pro-inflammatory microenvironment in COPD-insomnia comorbidity, characterized by innate immune dominance and adaptive immune suppression.

## 3. Discussion

This study provides strong genetic evidence for a unidirectional causal relationship between insomnia and COPD, building on previous studies on insomnia and COPD [23]. Previous studies have suggested a possible association between insomnia and COPD, and in our study when insomnia was modeled as an exposure variable, the significant positive correlation between them suggests that genetic susceptibility to insomnia independently increases the risk of COPD, whereas reverse MR analysis showed no causal effect of COPD on insomnia [24]. These findings support prior observational studies by mitigating confounding biases and positioning insomnia as a modifiable risk factor for COPD rather than a secondary consequence. Mechanistically, chronic sleep disruption may exacerbate systemic inflammation, oxidative stress, and autonomic dysfunction—whose pathways are implicated in airway remodeling and COPD progression. The absence of reverse causality aligns with the hypothesis that COPD-related symptoms (e.g., nocturnal dyspnea) may impair sleep quality without directly affecting genetic predisposition to insomnia. Clinically, these results underscore the importance of integrating sleep health into COPD prevention strategies, as early identification and management of insomnia could mitigate respiratory morbidity [25].

Building on the unidirectional causal link between insomnia and COPD, we elucidated the molecular mechanisms of this comorbidity. A total of 230 commonly dysregulated genes in GEO datasets, including both upregulated and downregulated profiles, were identified. PPI network analysis of the DEGs revealed enrichment in the following modules: anti-inflammatory and immunosuppression (e.g., IL10 and TNFAIP3), pro-inflammatory and immune cell activation (e.g., IL7R, C5AR1, FPR1 and LYN), chemokines and cell migration (e.g., CXCR4 and CCR7), transcriptional regulation and signal transduction (e.g., IRF1, NFKBIA and HIF1A), tumor suppression and cell cycle regulation (e.g., PTEN and PTGS2), metabolism and lipid transport (e.g., SCARB1), and cytoskeleton and cell motility (e.g., PLEK). This suggests that systemic inflammation and neuroimmune crosstalk may mediate the insomnia–COPD axis. Pivotal protein, such as IL10, PTGS2, and TNFAIP3, emerged as central nodes, highlighting their key role in a dual regulatory mechanism. These genes are involved in airway inflammation while the regulation of the sleep–wake cycle through cytokine-mediated hypothalamic pathways. These findings are highly consistent with our MR results, thus supporting the role of chronic inflammation in inducing the development of COPD [26]. In addition, sleep deprivation may exacerbate chronic inflammation, thereby disrupting sleep homeostasis in a vicious cycle [27]. The ssGSEA results further corroborate this inflammatory axis, demonstrating a convergent infiltration pattern of pro-inflammatory innate immune cells (neutrophils and macrophages) in both conditions. This cellular evidence aligns with the transcriptomic signatures of NF-κB activation and cytokine production, suggesting that TNFAIP3 may function as a key regulator at the interface of chronic inflammation and immune cell recruitment.

Moreover, this study systematically bridges epidemiological causality (MR-derived OR = 2.04, *p* = 0.011) with molecular convergence through four synergistic analytical layers: (1) 230 co-dysregulated genes at the COPD–insomnia interface, demonstrating immune–inflammatory axis dominance; (2) TNFAIP3 as a redox rheostat bridging NF-κB signaling and circadian rhythm pathways; (3) TNFAIP3, CXCR4, and PTGS2 as triple diagnostic signatures; and 4) TNFAIP3-targeting agents with dual anti-inflammatory and neuroregulatory abilities [28]. These findings advance a paradigm shift in comorbidity management: rather than treating COPD and insomnia as distinct entities, we identified therapeutic agents targeting their common TNFAIP3-centered interactome to disrupt the inflammation-sleep disruption cycle through single-agent polypharmacology.

Building upon the above findings, we implemented a systems pharmacology approach to repurpose dual-efficacy therapeutics. To our knowledge, this is the first study to provide evidence that TNFAIP3-targeted agents could mitigate COPD progression (via NF-κB suppression) and insomnia pathophysiology (via cytokine-mediated SCN modulation) [29].

Nevertheless, this study had some limitations. First, all data in the analyses were derived from European and Asian Biobanks, thereby limiting the generalizability of our findings to global populations; moreover, residual pleiotropy (MR-Egger intercept *p* = 0.38) cannot fully exclude the confounding effects of unmeasured cardio-metabolic traits. Second, this study had target selection bias. Stringent DEG thresholds (log2FC > 1.0) may have excluded epigenetically silenced regulators of COPD–insomnia crosstalk (e.g., circadian repressors PER/CRY). Third, TNFAIP3’s dual role in NF-κB suppression and circadian regulation should be confirmed in conditional knockout models assessing lung–brain axis disruption, filling some mechanistic knowledge gaps. Lastly, there are certain therapeutic repurposing caveats. DSigDB’s neuropsychiatric drug skew reflects historical research bias rather than biological relevance, necessitating in vitro validation (e.g., TNFAIP3 ubiquitination assays in BEAS-2B bronchial cells and primary suprachiasmatic neurons) and in vivo efficacy testing (berbamine in elastase/CLOCKΔ19 murine models with spirometry-polysomnography phenotyping). These limitations underscore that although multi-omics convergence identifies plausible comorbidity therapeutics, clinical translation demands mechanism-guided trials bridging computational predictions with pathophysiological reality [30].

Building on TNFAIP3’s mechanistic duality as a ubiquitin-editing hub for NF-κB (COPD pathogenesis) and circadian rhythm modulation (insomnia pathophysiology), its therapeutic exploitation warrants multi-modal drug engineering. First, small-molecule optimization can be performed through structure–activity refinement of berbamine analogs (e.g., C7-hydroxyl substitution to enhance TNFAIP3 OTU domain binding while reducing hERG channel affinity). Second, representing antibody-based biologics, TNFAIP3-stabilizing nanobodies can be developed to prolong its anti-inflammatory activity via reduced proteasomal degradation. Third, TNFAIP3 agonists (e.g., bardoxolone derivatives) can be conjugated with antibacterial agents via pH-sensitive linkers for lung–brain axis targeting. Lastly, using CRISPR-activation systems, LNP-encapsulated saRNA constructs can be generated to amplify endogenous expression in alveolar macrophages and suprachiasmatic nucleus neurons [31]. Moreover, the observed immune infiltration patterns—specifically the enrichment of neutrophils and macrophages—provide a plausible cellular mechanism through which TNFAIP3 dysregulation may perpetuate systemic inflammation. As a negative regulator of NF-κB signaling, TNFAIP3’s impaired function could lead to uncontrolled activation of these innate immune populations, creating a feed-forward loop of inflammation that affects both pulmonary and neurological tissues.

In the context of COPD co-occurring with mental disorders, inflammatory mechanisms are considered one of the key factors. Research indicates that crocin suppresses inflammation via the PI3K/Akt signaling pathway, thereby alleviating COPD-induced depressive symptoms [32]. This correlates with TNFAIP3’s role in regulating inflammation, suggesting Berbamine may exert effects through analogous mechanisms by influencing TNFAIP3 expression or function, potentially contributing to the treatment of COPD co-occurring with insomnia. In patients with major depressive disorder, TNFAIP3 mRNA levels were significantly correlated with psychological anxiety symptoms [33]. This further supports the potential regulatory role of TNFAIP3 in psychiatric disorders, suggesting that Berbamine may improve psychological symptoms associated with COPD by influencing TNFAIP3.

In exploring the mechanism of action of Berbamine on TNFAIP3 and its potential application in treating the co-morbidity of chronic obstructive pulmonary disease (COPD) and insomnia, we require a thorough understanding of TNFAIP3’s role in cellular survival and inflammatory regulation. As an ubiquitin-modifying enzyme, TNFAIP3 has been demonstrated to exert significant anti-inflammatory effects across multiple cell types, supporting cellular survival by restricting the mTOR signaling pathway and promoting autophagy [34].

Firstly, the mechanism of action of TNFAIP3 in CD4 T cells offers a significant perspective. Research indicates that TNFAIP3 enhances the survival capacity of CD4 T cells by restricting the mTOR signaling pathway and promoting autophagy [34]. This mechanism may exert a similar protective effect in the comorbidity of COPD and insomnia, as COPD is frequently accompanied by chronic inflammation, while insomnia may be associated with immune system dysregulation.

Furthermore, the anti-inflammatory effects of TNFAIP3 have been demonstrated in other cell types. For instance, within Leydig cells, TNFAIP3 protects against the inflammatory microenvironment by inhibiting the p38 MAPK signaling pathway and upregulating CEBPβ expression, thereby promoting testosterone synthesis [35]. This anti-inflammatory mechanism may play a role in the pathophysiology of COPD, given the disease’s close association with chronic inflammation.

In terms of mental health, TNFAIP3 expression levels are significantly correlated with psychological anxiety symptoms in patients with depression [33]. This suggests that TNFAIP3 may influence depressive symptoms co-occurring with COPD by regulating inflammatory responses. Furthermore, studies indicate that inhibiting inflammatory signaling pathways can ameliorate COPD-induced depressive symptoms [32], further supporting TNFAIP3’s potential role in modulating inflammation and mental wellbeing.

In summary, Berbamine may influence the co-morbid mechanisms of COPD and insomnia by regulating the expression or function of TNFAIP3. TNFAIP3 may play a pivotal role in the treatment of these conditions by restricting the mTOR signaling pathway, promoting autophagy, and inhibiting inflammatory signaling pathways. Future research should further explore the specific regulatory mechanisms of Berbamine on TNFAIP3, with the aim of providing novel therapeutic strategies for the co-morbid treatment of COPD and insomnia.

It is noteworthy that our transcriptomic and drug repositioning analyses, though derived from general adult cohorts, reflect pathophysiological processes—such as chronic inflammation, oxidative stress, and immune senescence—that are exacerbated in the elderly. Aging is associated with dysregulated immune responses and impaired redox homeostasis, which may amplify the TNFAIP3-centered mechanisms identified herein. Future studies should prioritize validation in aged murine models or human cohorts with stratified age groups to elucidate geriatric-specific therapeutic windows.

In the treatment of chronic obstructive pulmonary disease (COPD) complicated by insomnia, the mechanisms of action of TNFAIP3 and Berbamine have attracted considerable attention. Therapeutically, Berbamine, as a natural alkaloid, demonstrates significant anti-inflammatory and immunomodulatory effects. Research indicates that Berbamine can reduce pulmonary inflammatory responses by inhibiting NLRP3 inflammasome activation, offering potential therapeutic benefits for COPD. Furthermore, TNFAIP3, acting as a negative regulator, exerts a protective role in COPD inflammation by modulating inflammatory signaling pathways and reducing the release of inflammatory mediators [36]. In summary, TNFAIP3 and Berbamine hold significant research value in the treatment of insomnia associated with COPD. By modulating inflammatory responses and improving sleep quality, these two substances may offer a novel therapeutic strategy for COPD patients. Future studies should further investigate their specific mechanisms of action and clinical application potential, with the aim of providing additional treatment options for patients with COPD-associated insomnia.

Notably, the overproduction of reactive oxygen species in COPD patients not only damages airway epithelia, but also impairs the function of suprachiasmatic nucleus, a master regulator of sleep–wake cycles [37]. Through its ubiquitin-editing activity, TNFAIP3 modulates both NF-κB–driven inflammation and NRF2-mediated antioxidant responses, representing a potential therapeutic target for COPD–insomnia comorbidity [38].

## 4. Materials and Methods

### 4.1. Mendelian Randomisation

We implemented a bidirectional two-sample Mendelian randomization (MR) framework in accordance with the STROBE-MR guidelines [39]. Genetic data for insomnia were derived from the European-ancestry GWAS by Elsworth et al. (*N* = 462,341; GCST90012345), and chronic COPD instruments were from Sakaue et al.’s Biobank Japan study (Ncase = 468,475/Ncontrol = 454,945; GCST000876). The screening process adhered to the following core assumptions of MR analysis: (1) relevance (GWAS significance threshold *p* < 5 × 10^−8^), (2) independence (LD clumping via PLINK2: r^2^ < 0.001, 10 Mb window), and (3) exclusion restriction (Steiger filtering *p* < 0.01). Weak instrument bias was mitigated through variance-adjusted F-statistics (>10). Causal estimates were generated via inverse-variance weighted (IVW) regression, alongside sensitivity analyses including MR-Egger intercept testing (pleiotropy = 0.32), weighted median estimation, and Cochran’s Q heterogeneity assessment (I^2^ = 18%, PQ = 0.24) [40].

### 4.2. Targets of COPD and Insomnia

The Gene Expression Omnibus (GEO, http://www.ncbi.nlm.nih.gov/geo/ accessed on 14 October 2024) database was systematically searched for keywords “chronic obstructive pulmonary disease” and “insomnia.” A collection of COPD and insomnia related Gene Expression profile chip data. Preprocessing of the original data, including standardization, correction, and gene-name annotation, was performed in R (v4.1.2). The R language-based limma package (v3.44.0) was used for the analysis of DEGs. The upregulated and downregulated DEGs in each set of chip data were screened based on the following thresholds: log2|fold change| > 1.0 and *p* < 0.05. Integration of transcriptomic data from COPD and insomnia cohorts revealed 230 consistently dysregulated genes across both conditions.

### 4.3. GO/KEGG Enrichment Analysis

We performed enrichment analyses of consistently dysregulated genes [41]. To elucidate the biological significance of shared genes between COPD and insomnia, Gene Ontology (GO) and KEGG pathway analyses were prioritized for terms related to antioxidant defense (e.g., response to oxidative stress, glutathione metabolism) and inflammasome activation (e.g., NF-κB signaling and NLRP3 complex) [42]. TNFAIP3-centric networks were searched for reactive oxygen species (ROS)-related interactions using ReactomePA (v1.42.0) [43]. GO terms were categorized into the following domains: biological process (BP), molecular function (MF), and cellular component (CC). Terms and pathways with thresholds of *p* < 0.05 and Q < 0.05 (adjusted for false-discovery rate [FDR]) were deemed significant [44].

### 4.4. PPI Network Analysis

To delineate functional interplay among shared genes, a PPI network was constructed for the 230 consistently dysregulated genes using the STRING database (v12.0) [45]. Node centrality was quantified by degree values, with hub genes defined as those exceeding the 90th percentile of degree distribution. Visualization and modular analysis were performed in Cytoscape (v3.10.0) using the degree algorithm to identify densely interconnected clusters [46].

### 4.5. WGCNA

To identify gene clusters associated with COPD and insomnia, WGCNA was applied to transcriptomic datasets GSE148004 (COPD) and GSE208668 (insomnia) using the WGCNA package (v1.73) in R (v4.0.3) [47]. Raw expression matrices were preprocessed by removing outliers (sample hierarchical clustering), normalizing for batch effects (sva package, 15 October 2024), and filtering low-variance genes (retaining the top 75% by median absolute deviation). A signed network was constructed with a soft-thresholding power (β = 12 for COPD, β = 10 for insomnia) selected via scale-free topology criterion (R^2^ > 0.85). Dynamic tree-cutting (deepSplit = 2, minModuleSize = 30) identified 10 co-expression modules across both datasets, each of which was color-coded for visualization. Module–trait relationships were quantified by correlating module eigengenes (first principal component) with clinical phenotypes (e.g., COPD severity and insomnia index) using Pearson’s correlation (*p* < 0.05). A heatmap was generated to visualize module–phenotype associations with hierarchical clustering (Euclidean distance and complete linkage), and modules were grouped according to functional similarity.

### 4.6. LASSO Regression Analysis

LASSO analysis is a regression-based algorithm that allows for the presence of a large number of covariates in the model and has the unique feature of minimizing the sum of the absolute regression coefficients [48]. LASSO analysis of candidate hub genes was performed, and Differential Expression Analysis using the “glmnet” package (accessed on 15 October 2024) of R software (version 4.4.0). Three overlapping features and three variables were identified as diagnostic biomarkers for COPD and insomnia [49].

### 4.7. Drug Repositioning Analysis

To identify potential therapeutic agents targeting shared COPD–insomnia mechanisms, gene signature-based drug repositioning was performed using the Drug Signatures Database (DSigDB) via the Enrichr platform (September 2023 release, https://maayanlab.cloud/Enrichr/ accessed on 15 October 2024). The DSigDB catalogs 22,527 gene–drug signatures derived from transcriptomic and proteomic studies, enabling the systematic mapping of disease-associated genes to pharmacological modulators. Gene sets (e.g., TNFAIP3-centric network) were uploaded to Enrichr, and DSigDB enrichment was conducted using default hypergeometric test parameters. Drug-gene associations were filtered according to adjusted *p* < 0.05 and normalized combined score > 50, where the combined score integrated gene overlap significance (P), signature concordance (directionality of gene-expression changes), and pharmacological evidence (dose–response and multi-omics validation) [50].

### 4.8. Molecular Docking Analysis

Molecular docking was performed using the AutoDock Vina (version 1.1.2) (accessed on 15 October 2024) software. The top 10 drugs (according to combined score) predicted by the Enrichr database with strong binding affinities were selected. We utilized the results of compound-target construction to determine whether there is any interaction between TNFAIP3 and the predicted small-molecule drugs [51]. TNFAIP3’s structure was downloaded from the PDB database [https://www.rcsb.org/, accessed on 15 October 2024, PDB ID:2vfj and converted to the PDB format. Protein residues were completed by Chimera (v1.18), and missing atoms were supplied by Pymol (v3.1). The structures of the small-molecule compounds were downloaded from the PubChem database (https://pubchem.ncbi.nlm.nih.gov/, accessed on 23 February 2025) and converted to the sdf format, which was transformed using OpenBabel (accessed on 15 October 2024) to obtain Mol2 files [46]. The receptor file was imported into AutoDockTools (v1.5.6) and converted to the pdbqt format. After removing solvents, heterogeneous molecules and full hydrogen were added to the receptor. The ligand was preprocessed using AutoDockTools by adding all hydrogens and exporting it as a pdbqt file. Based on the docking results, the complexes were visualized using the Pymol [52].

### 4.9. Immune Infiltration Analysis by Single-Sample GSEA (ssGSEA)

To characterize the immune microenvironment in COPD and insomnia, we performed single-sample gene set enrichment analysis (ssGSEA) using the GSVA package (v1.5) in R. This method quantifies the relative abundance of 28 distinct immune cell types within each sample’s transcriptomic profile. Normalized enrichment scores (NES) were calculated for each immune cell type, representing their relative infiltration levels. Differences in immune cell abundance between disease and control groups were visualized using heatmaps, with hierarchical clustering to reveal patterns of immune dysregulation specific to each condition.

## 5. Conclusions

This study revealed TNFAIP3’s dual role as a regulator of redox homeostasis and a suppressor of inflammatory signaling in COPD–insomnia comorbidity. Moreover, natural small molecules, with antioxidant and anti-inflammatory properties, were identified, highlighting the paradigm shift toward multitarget therapeutics that concurrently mitigate oxidative lung injury and restore circadian integrity—a strategy aligned with the emerging field of redox chronotherapy. The therapeutic blueprint derived from our systems pharmacology analysis—combining circadian rhythm stabilization with A20 pathway modulation—heralds a new era in comorbidity management, where a network medicine approach guides multi-organ intervention.

Our findings underscore the potential of TNFAIP3-targeted strategies in mitigating COPD–insomnia comorbidity, particularly in the elderly, who are often underrepresented in mechanistic studies yet overrepresented in clinical prevalence. The integration of geriatric-focused biomarkers (e.g., senescence-associated secretory phenotype factors) in future multi-omics frameworks could further personalize therapeutic approaches for this high-risk population.

Our findings indicate the role of TNFAIP3 as a redox rheostat, bridging NF-κB–driven inflammation and NRF2-mediated antioxidant responses. As the top-ranked compound in molecular docking analysis, berbamine not only inhibits TNFAIP3’s deubiquitinase activity, but also shares structural homology with known NRF2 activators (e.g., sulforaphane). This suggests a dual-action mechanism: suppressing IL-1β release while enhancing endogenous antioxidant defenses by modulating the KEAP1/NRF2 axis. Considering the relationship between systemic oxidative burden and poor sleep quality, TNFAIP3-targeted antioxidants could disrupt the ROS–inflammation–insomnia cycle. Therefore, future studies should assess the efficacy of these compounds in reducing 8-OHdG (a biomarker of oxidative DNA damage) levels in COPD patient sera improving polysomnography metrics.

## Figures and Tables

**Table 1:** LASSO regression analysis results.

Gene	Coef
*CXCR4*	1.05523161222664
*IRF1*	0.279011336498418
*TNFAIP3*	0.4521141338732

**Table 2:** Drug prediction results.

Term	*p*	Adjusted *p*	OR	Combined Score
BERBAMINE CTD 00001312	5.499852259717758 × 10^−4^	0.012660698449782595	19,989.00	150,029.8210287992
tolbutamide CTD 00006903	5.999842544083076 × 10^−4^	0.012660698449782595	19,988.00	148,283.11962915692
PIPERONYL BUTOXIDE CTD 00006567	5.999842544083076 × 10^−4^	0.012660698449782595	19,988.00	148,283.11962915692
fenthion CTD 00005965	8.999788927950273 × 10^−4^	0.012660698449782595	19,982.00	140,136.54844084635
trimipramine MCF7 UP	9.999772468562472 × 10^−4^	0.012660698449782595	19,980.00	138,017.40508704726
cloperastine MCF7 UP	0.0010499764455398322	0.012660698449782595	19,979.00	137,035.71221916509
ticlopidine MCF7 UP	0.001099975657412352	0.012660698449782595	19,978.00	136,099.47030191717
clonidine CTD 00005689	0.001249973366379199	0.012660698449782595	19,975.00	133,525.54486996148
lasalocid PC3 UP	0.001249973366379199	0.012660698449782595	19,975.00	133,525.54486996148
econazole MCF7 UP	0.0012999726251831402	0.012660698449782595	19,974.00	132,735.46073187632

**Table 3:** Molecular Docking Analysis result.

Molecule	Free Energy (kcal/mol)	Molecule	Free Energy (kcal/mol)
Berbamine	−9.25	Fenthion	−6.08
Tolbutamide	−8.68	Trimipramine	−5.98
Ticlopidine	−8.02	Clonidine	−5.76
Econazole	−7.1	Piperonyl	−4.93
Cloperastine	−6.58	Lasalocid	−1.43

## Data Availability

RNA expression data can be found at https://www.ncbi.nlm.nih.gov/geo/ accessed on 14 October 2024, and all data generated in this study are included in this article and its Supplementary Materials.

## Supplementary Materials

The following supporting information can be downloaded at: https://www.mdpi.com/article/10.3390/ijms262010227/s1.

## Abbreviations

The following abbreviations are used in this manuscript:

COPD	Chronic obstructive pulmonary disease
MR	Mendelian randomization
PPI	Protein–protein interaction
WGCNA	Weighted gene co-expression network analysis
