# Supplementary material for: Berbamine Targets TNFAIP3: A Bioactive Compound Alleviates Oxidative Stress and Inflammation in the Comorbidity of Insomnia and Chronic Obstructive Pulmonary Disease Through Multi-Omics Integration
Source: Int J Mol Sci. 2025 Oct 21;26(20):10227. doi: 10.3390/ijms262010227 (PMC12563523; doi:10.3390/ijms262010227)
Supplement: Supplementary file 1 [file ijms-26-10227-s001.zip › ijms-3843205-supplementary.pdf]

# Supplement material

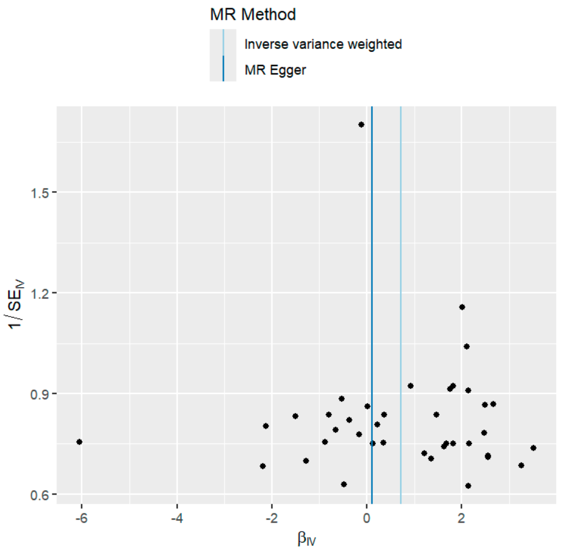


**Figure S1.** Mendelian Randomization Scatterplot.

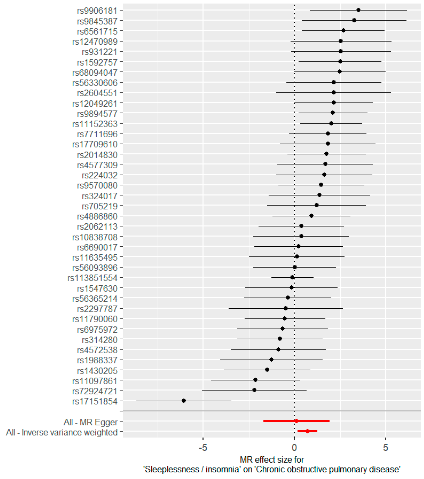


**Figure S2.** Verification of the Reliability of Mendelian Randomization Results.

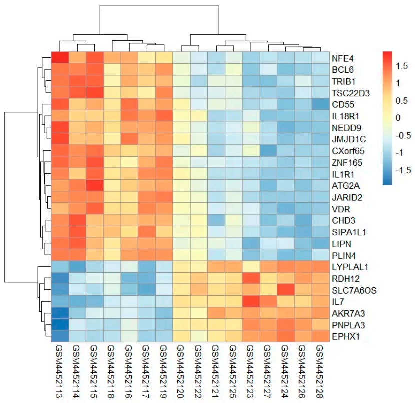

**Figure S3.** Heatmap of COPD.

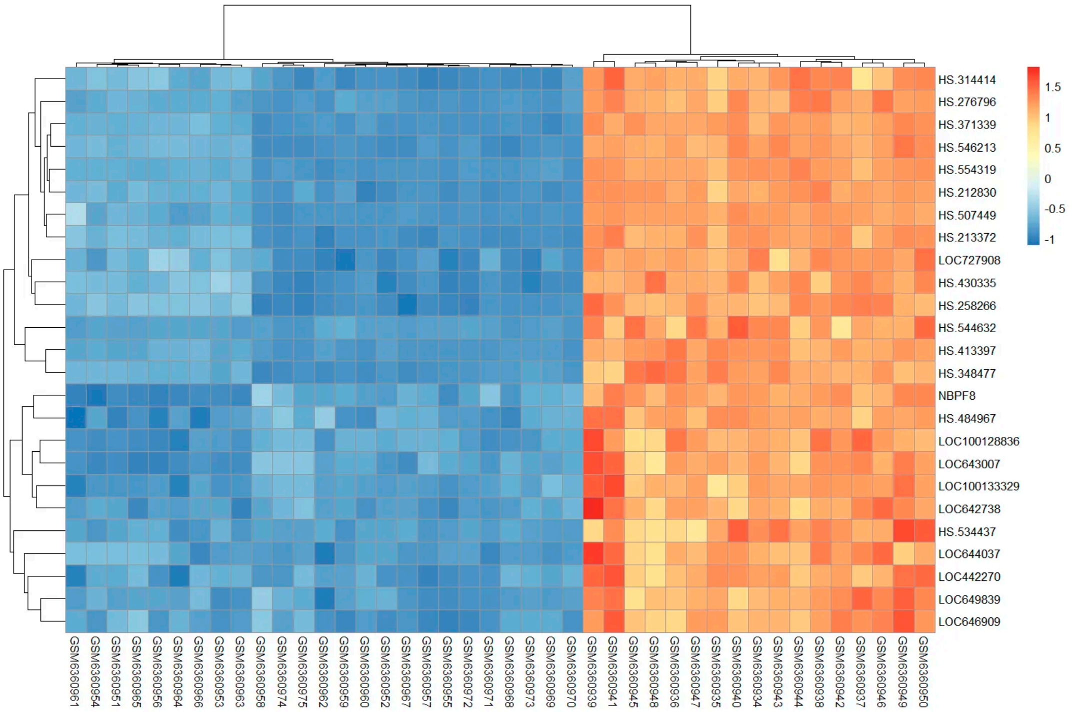


**Figure S4.** Heatmap of insomnia.

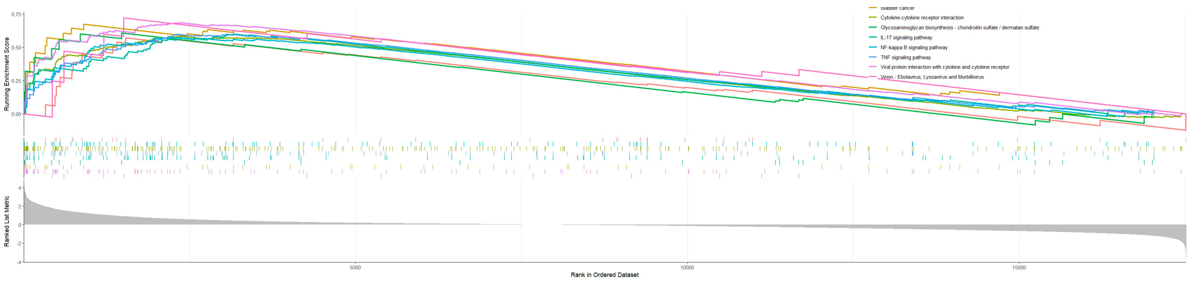


**Figure S5.** GSEA Analysis of COPD.

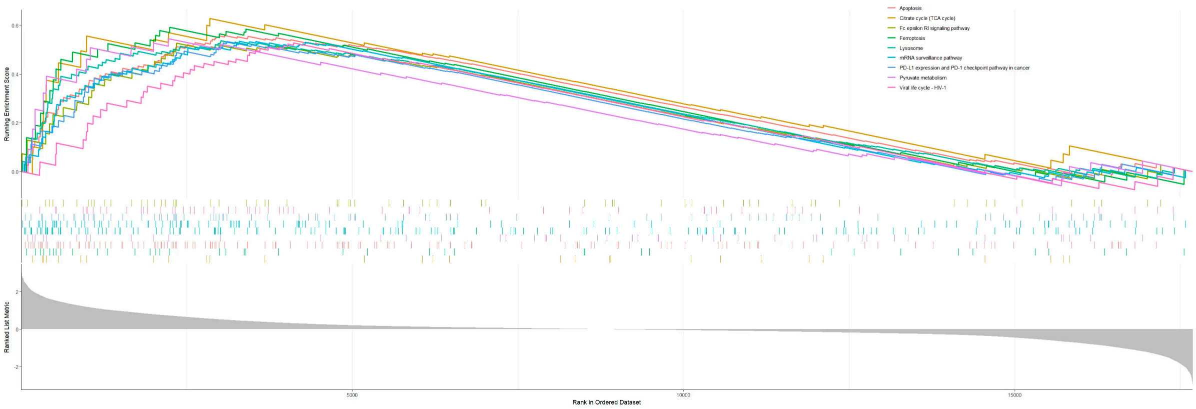


**Figure S6.** GSEA Analysis of insomnia.

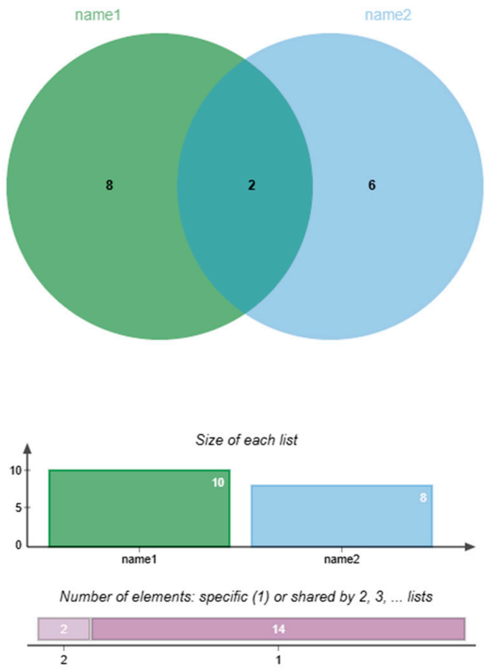


**Figure S7.** The overlap between Berbamine's drug targets and the targets identified in this study.
